# Middlesex Hospital

**Published:** 1831-06

**Authors:** 


### HOSPITAL REPORTS.

### MIDDLESEX HOSPITAL.

### *Ulcerative Disease of the Rectum removed by Excision, by* Mr. Mayo.

We gave the report of this very interesting case in our number for April, p. 319. We saw the woman a few days ago, and found her much improved in every respect: the general health is better, and the sufferings she experienced from the disease have been entirely removed by the operation. The most important point, however, to notice is, that she can perfectly retain solid fæces. As might be expected, when she is under the influence of purgative medicine, the contents of the bowels are discharged rather quickly; but it seems far from impossible that, in time, the firm cicatrix at the wound, assisted by the action of the muscular fibres of the bowel itself, will form a sufficient substitute, on all occasions, for the sphincter muscle, which has been removed.

### *Strangulated Hernia; Entero-Epiplocele; the Omentum tied, and removed.*

Eliza Sawyer, æt. forty-eight, was admitted into the Middlesex Hospital, on the evening of Monday, 18th of April. She had been subject, for several years, to crural hernia in the right groin: she had worn a truss, but it had fitted imperfectly, and something, she thought, had always remained down since the commencement of the rupture. On the Wednesday preceding her admission, she had been attacked with vomiting and disorder of the bowels: on the same evening she observed that the swelling in the groin was enlarged considerably, and had become uneasy and painful. She had no sleep that night. The following morning, she took an ounce of castor oil, and a few hours afterwards an ounce of salts: these remedies produced no action of the bowels. She had no sleep on the nights of Thursday, Friday, Saturday, and Sunday; nor did the bowels act.

When Mr. Mayo saw her a short time after her admission, there was stercoraceous vomiting, and the countenance was worn and anxious; the belly hard, and rather tender on pressure. The tumour felt of a doughy consistence, as if it consisted partly of omentum: pressure upon it gave pain. She had been in the warm bath; a purgative enema had been administered, and some attempts had been made to return the rupture by the taxis.

Mr. Mayo proceeded with little delay to perform the customary operation: the sac was laid open; the stricture, which was exceedingly close, divided; and a knuckle of small intestine, which was only dark coloured, was returned into the belly. It remained to dispose of a thickened portion of omentum, which had lain before the intestine in the sac, and which, it appeared, could not be returned into the abdomen without being greatly bruised, or without considerable additional enlargement of the stricture.

In deliberating what should be done with this portion of omentum, Mr. Mayo briefly adverted to a case, that had occurred in the hospital, in which he had removed a portion of omentum, and tied with single threads the vessels which bled, and returned the part into the belly: this patient did well, but the hernia returned, and the patient had again to wear a truss. Mr. Mayo mentioned also another case, that had likewise occurred in the hospital, in which, after returning the intestine, he had left a portion of thickened omentum unreturned in the sac: the patient did well, the wound healed kindly, but the rupture returned. In another instance, Mr. Mayo had adopted a different plan. In a case of inguinal hernia, occurring in a gentleman, in December last, a large mass of thickened omentum had to be dealt with: in this instance, Mr. Mayo had passed a double ligature through the neck of the mass of protruding omentum, and having tied one half the ligature upon either half of the root of the mass, he had cut off the mass half an inch from the ligatures. The patient did prosperously: there has been no recurrence of hernia in this case, and the patient is persuaded, from the feeling of strength and security in the groin, of the want of which, while he was liable to hernia before the operation, he was always conscious, that the passage is now blocked up and secured by the adherent extremity of the omentum.

The practice last described Mr. Mayo adopted in the case of Eliza Sawyer.

After the operation, the patient, exhausted by several days of suffering, complained of being faint and low: some warm wine and water was given to her, which she drank eagerly. She passed a tolerable night. The bowels acted spontaneously the next day. The day following she complained of slight tenderness of the belly: the bandage, which had been applied after the operation, was therefore removed, the tightness of which might have caused or contributed to the uneasiness she complained of, and a poultice was applied to the groin; fourteen leeches were applied, besides, as a measure of precaution, to the abdomen; and two grains of calomel, and three of James's powder, administered at night, with an opening draught the following morning.

The progress of the case has been perfectly favorable: the ligatures came away from the omentum on the ninth day; the wound is now healed, (10th May.)

### *Fractured Ribs; Empyema; Paracentesis Thoracis.*

John Berryman, æt. fifteen, was admitted into the Middlesex Hospital, under the care of Mr. Mayo, on the 21st of March. He had been kicked on the left side by a horse, and the fourth and fifth ribs had been fractured. There was some emphysema upon the side and back, but no blood was expectorated: he complained of pain in the side on breathing. He was bled on his admission, and again on the following day. This, with suitable medicines, and bandaging, relieved the pain in breathing and coughing: the emphysema diminished.

On the 25th, however, he had a smart rigor, followed by heat of skin, frequent sharp pulse, pain in the side, and uneasy breathing: he was bled on that day, and again the next. The inflammatory symptoms now diminished, but the breathing became more distressed.

On the 30th, it was evident that he breathed only with the right side of the chest; the heart, at the same time, could not be felt upon the left side of the chest, but beat strongly in the epigastrium, and upon the right side. No fulness, however, could be observed between the ribs of the left side, and the breathing, though frequent and uneasy, did not labour enough to require any immediate attempt to be made to relieve it.

He was carefully watched, but no decisive change, in any respect, had taken place before the 14th of April, although the boy was gradually becoming weaker, and his breathing more difficult in the interval. On the 14th, distinct fluctuation could be felt between the fractured ribs; the intercostal space was full and prominent. Mr. Mayo, therefore, made an opening into the chest at this part. The skin was divided for an inch, but the opening through the pleura was oblique, and only large enough to admit the cannula of a small trochar. Eighteen ounces of pus, sufficiently thick, but pale in colour, came away: a considerable quantity of cotton wool was then placed upon the wound, with the ordinary rib roller to support it. When the dressing was changed in the evening, it was found soaked with the pus, which had continued to flow.

The following day, the boy expressed himself considerably relieved in his breathing. Matter continued to flow away in the dressings for several days: the discharge then suddenly diminished, and the boy's breathing became more uneasy: the wound in the chest was then enlarged, and now air for the first time distinctly entered the chest during inspiration; but at the same time the matter again flowed abundantly, and the patient found himself again relieved.

In a few days after this, the heart was no longer to be felt on the right side of the chest, or in the epigastrium. The boy's appetite returned, his tongue became perfectly clean, and his sleep easy and refreshing. The discharge from the chest is now daily diminishing. The boy is greatly improved in strength, and appears likely to do perfectly well. (May 17th.)

### *Removal of the Breast for Scirrhus.*

Elizabeth Tottleby, ætat. fifty-one, was admitted into the Middlesex Hospital, under the care of Mr. Mayo, on the 23d of March, with scirrhus of the left breast. The tumour, which had been a year in forming, and was of the size of an egg, occupying almost all the gland of the breast, adhered very loosely to the pectoral muscle. There was no enlargement or tenderness in the axilla or above the clavicle. The breast was removed on the 25th: it had the ordinary appearance of a schirrous structure. Mr. Mayo remarked, that the membranous texture of the surrounding fat, which was divided, was hard and gristly; and that he augured ill of the final result of the operation.

The wound has healed; but there has occurred, subsequently, a suspicious fulness, with shooting pain at one or two points near the cicatrix: these symptoms have been relieved as yet by the application of leeches.

There is another patient in the Cancer ward, by name Mary Smith, whose breast Mr. Mayo removed in October last. She is seventy-four years of age: the scirrhus was of great size, a small part of the surface had already begun to slough, and two glands were enlarged in the axilla. Mr. Mayo determined to operate in this case, for the following reasons: As the tumor had begun to slough, it was probable that the patient, if nothing were done, would in the course of a month be afflicted with a large open cancer; but, as the integuments of the breast at every part but one were healthy, it was likely that, if the whole tumor were removed, the wound would heal kindly, and the disease be at least temporarily got rid of. Again, as the disease had been six years in forming, and the age of the patient so advanced, it was likely that the return of the disease would be naturally extremely slow, and perhaps would admit of being additionally retarded by judicious management.

These anticipations have been completely realized: the wound made by the operation healed readily, and now the only mark of the return of the complaint is a hardness of one end of the cicatrix, and some tenderness, and trifling hardness and fulness, in the axilla, from which the glands, which were diseased, were removed at the same time with the breast. The tenderness and hardness in the cicatrix and axilla began to shew themselves in December: to remedy these symptoms, leeches were frequently and profusely applied, with hot fomentations and poultices; and as often as the tenderness and hardness, accompanied with shooting pains, have recurred, the same means have been again employed with success, and the advance of the disease has been arrested.

In order that the patient may not be generally reduced by this repeated local depletion, she is allowed full diet and wine. Her appearance is now very hale and healthy, and she exists in comfort and enjoyment, instead of being the victim of a painful and dreadful disease, which certainly would have been her fate, if the operation had not, perhaps against the ordinary rules, been performed.

